# Coronary Perfusion Pressure during Antegrade Cardioplegia in On-Pump CABG Patients

**DOI:** 10.21470/1678-9741-2017-0035

**Published:** 2017

**Authors:** Jackson Brandão Lopes, Carlos Cezar Monteiro dos Santos Júnior

**Affiliations:** 1 Faculdade de Medicina da Bahia da Universidade Federal da Bahia (FMB-UFBA), Salvador, BA, Brazil.

**Keywords:** Heart Arrest, Induced, Blood Pressure, Cardioplegic Solutions/*Administration & Dosage, Coronary Artery Bypass, Coronary Artery Disease

## Abstract

**Objective:**

The aim of this study was to investigate whether aortic tension estimated by palpation and cardioplegia infusion line pressure provide results equivalent to those obtained with direct aortic intraluminal pressure measurement.

**Methods:**

Sixty consecutive patients who underwent coronary artery bypass graft surgeries with extracorporeal circulation were analyzed. Sanguineous cardioplegic solution in a ratio of 4:1 was administered using a triple lumen antegrade cannula. After crossclamping, cardioplegia was infused and aortic root pressure was recorded by surgeon (A) considering the aortic tension he felt in his fingertips. At the same time, another surgeon (B) recorded his results for the same measurement. Concomitantly, the anesthesiologist recorded intraluminal pressure in the aortic root and the perfusionist recorded delta pressure in cardioplegia infusion line. None of the participants involved in these measurements was allowed to be informed about the values provided by the other examiners.

**Results:**

The Bland-Altman test showed that a considerable variation between aortic wall tension was found as measured by palpation and by intraluminal pressure, with a bias of -9.911±18.75% (95% limits of agreement: -46.7 to 26.9). No strong correlation was observed between intraluminal pressure and cardioplegia line pressure (Spearman's r=0.61, 95% confidence interval 0.5-0.7; *P*<0.0001).

**Conclusion:**

These findings reinforce that cardioplegia infusion should be controlled by measuring intraluminal pressure, and that palpation and cardioplegia line pressure are inaccurate methods, the latter should always be used to complement intraluminal measurement to ensure greater safety in handling the cardioplegia circuit.

**Table:** 

Abbreviations, acronyms & symbols	
CABG	= Coronary artery bypass graft
CPB	= Cardiopulmonary bypass
EACTS	= European Association for Cardio-Thoracic Surgery
EAPCI	= European Association for Percutaneous Cardiovascular Interventions)
ESC	= European Society of Cardiology
LITA	= Left internal thoracic artery
LOA	= Limits of agreement
SUS	= Sistema Único de Saúde (Brazilian public health system)

## INTRODUCTION

Ischemic heart disease is a major cause of death in Brazil. In 2010, there were 99,955 deaths from ischemic heart disease in the country, with a mortality rate of 52.4/100,000 population^[1]^.

In 2015, 20,198 coronary artery bypass graft (CABG) surgeries were performed with cardiopulmonary bypass (CPB) in Brazil^[2]^. Over the past few years, the profile of patients undergoing this surgery has changed, due to advances in percutaneous revascularization and improved clinical treatment^[3]^. Since the 1990s, studies have shown that today these patients are older, sicker, and have a higher risk than in the past^[4,5]^, which urges healthcare professionals to optimize care to reduce mortality rates^[5]^.

Myocardial protection is a critical point in on-pump CABG surgeries, and infusion pressure should be carefully monitored in order to avoid possible endothelial damages and reperfusion injuries^[6,7]^.

The estimation of infusion pressure during antegrade cardioplegia is based on the measurement of pressure in the initial portion of the aorta. The gold standard to determine aortic pressure is direct intraluminal measurement using a pressure monitoring line attached to the cardioplegia cannula. Currently, the Brazilian Unified Health System does not consider cannulas in compatible material list for CABG surgeries, thus making it difficult to monitor pressure during infusion of myocardial protection solution. In Brazil, the most used method to monitor aortic root pressure in surgeries is palpation with surgeon's fingertips, a method that may be considered subjective and vary according to surgeon's state of perception, experience, and sensitivity.

Hence, this study aimed to investigate whether aortic palpation and measurement of delta pressure in cardioplegia infusion line are equivalent to direct intraluminal measurement.

## METHODS

This prospective study shows the results of a quantitative exploratory-descriptive research with 60 consecutive patients who underwent elective on-pump CABG surgery from January 2014 to October 2014. The study was approved by the Human Research Ethics Committee of Centro Universitário do Leste de Minas Gerais/ UNILESTE - União Brasiliense de Educação e Cultura under no. 14142013.8.0000.5095.

### Eligibility Criteria

Patients of both sexes were included in this study. Selection criteria for CABG followed the norms established in the European Society of Cardiology (ESC), European Association for Cardio-Thoracic Surgery (EACTS) and European Association for Percutaneous Cardiovascular Interventions (EAPCI) Guidelines of Myocardial Revascularization 2010^[8]^. Patients were referred from the Department of Cardiovascular Surgery of Hospital Márcio Cunha / Fundação São Francisco Xavier, Ipatinga, Brazil, for routine surgeries. Patients indicated for on-pump CABG surgery who required other concomitant surgical procedures were excluded.

### Surgical Procedure

Myocardial protection was accomplished with cold (4°C) hyperkalemic blood (4:1) cardioplegia. The cardioplegic solution was administered using a triple lumen antegrade cardioplegic cannula (ATC011MV model, Edwards Lifesciences Inc., USA). One lumen was used for infusion, one for aspiration, and one for measurement of intraluminal pressure in the aortic root. This was measured by connecting the respective lumen to a Truwave PX 260 transducer (Edwards Lifesciences Inc., USA) that converts mechanical signals into electrical signals, and mean blood pressure was displayed in a DX 2020 multiparameter monitor (Dixtal, Brazil).

After heparinization and implementation of CPB using a Brizio^®^ membrane oxygenator (Nipro Medical Ltda., Brazil), the patient was kept at a systemic temperature of 34°C and the cardioplegia delivery system (Unique^®^, Nipro Medical Ltda., Brazil) was filled with blood and hyperkalemic crystalloid solution to remove the air from tubes and connectors. After occlusion of the ascending aorta, the left ventricle was initially suctioned by the cardioplegia cannula and the ascending aorta was perfused with the cardioplegia solution, which was infused at a flow rate of 300 ml/min until reaching a total volume of 10 ml/kg. Cardioplegia flow was adjusted according to aortic wall tension as estimated by the principal surgeon (A), using his fingertips. Aortic root pressure was recorded by surgeon (A) considering the aortic tension he considered adequate for coronary perfusion. At the same time, another surgeon (B), recorded his results for the same measurement. Concomitantly, the anesthesiologist recorded intraluminal pressure in the aortic root and the perfusionist recorded delta pressure in cardioplegia infusion line. None of the participants involved in these measurements was allowed to be informed about the values provided by the other examiners. This procedure was performed during every infusion of cardioplegia solution.

### Statistical Analysis

Data were analyzed using the Graphpad Prism 5 software, version 5D. Quantitative variables having a Gaussian distribution were described as mean ± standard deviation and those not having a Gaussian distribution were described as median (25^th^ percentile-75^th^ percentile). In turn, qualitative variables were described as absolute and relative frequencies. Data normality was tested using the Kolmogorov-Smirnov, Shapiro-Wilk, and D'Agostino tests. The degree of agreement between surgeons' measurements of aortic tension and between intraluminal pressures and measurements of aortic tension were assessed by the Bland-Altman test. The Spearman's correlation test was used to evaluate the association between intraluminal pressure and cardioplegia line pressure. Differences were considered significant when probability level was lower or equal to 0.05.

## RESULTS

### Overall Results

Demographic and comorbidity data are shown in Table 1. Intraoperative findings (Table 2) showed a mean number of grafts corresponding to the number of obstructed vessels, and anterior intraventricular artery was revascularized in all cases with left internal thoracic artery (LITA). In 10% of cases, the LITA was used to revascularize a second vessel. Saphenous vein graft was always the second more used graft rather than radial graft. Thirty-day mortality rate was 1.7%, and the incidence of atrial fibrillation and renal dysfunction was 8.3% and 5%, respectively. None of these cases evolved to need for renal replacement therapy. No cases of postoperative infection or stroke were observed (Table 3).

**Table 1:** Demography and comorbidity data.

Variable	n = 60
Age (years)	64±8#
Male, n (%)	51 (85%)
BMI	27±4.3#
Obese patients	16 (26.6%)
BMI < 25 kg/m^2^	19 (31.6%)
BMI from 25 to 29.9 kg/m^2^	25 (41.6%)
BMI from 30 to 34.9 kg/m^2^	13 (21.6%)
BMI > 35 kg/m^2^	3 (5%)
Hypertension, n (%)	51 (85%)
Smoking, n (%)	29 (48.3%)
COPD	-
Diabetes, n (%)	17 (28.3%)
Pacemaker, n (%)	1 (1.7%)
Previous AMI, n (%)	13 (21.6%)
Cerebrovascular disease, n (%)	8 (13.3%)
Stroke, n (%)	1 (1.7%)
Peripheral vascular disease, n (%)	2 (3.3%)
CRF requiring dialysis	-
Atrial fibrillation	-
ASA	-
P2Y12 inhibitor	-
Hemoglobin	13.5 ± 1.7#
Creatinine (mg/dL)	0.9 (0.8–1.1)$
LVEF	0.59±0.09#
≥ 0.5	89.8%
0.4 to 0.49	8.3%
0.3 to 0.39	1.7%
≤ 0.3	-
Pulmonary hypertension	
Moderate, n (%)	8 (13.3%)
Severe	-
Obstructed vessels	3.0±1.2#

BMI=body mass index; COPD=chronic obstructive pulmonary disease; AMI=acute myocardial infarction; CRF=chronic renal failure; ASA=acetylsalicylic acid; LV=left ventricle

# mean ± standard deviation;

$ median ± interquartile range (p25-p75)

**Table 2:** Intraoperative variables.

Variable	n = 60
Grafts	3.1 ± 0.9#
Arterial grafts	1 (1–1)$
Venous grafts	2 (1.5–3)$
LITA -> AIVA, n (%)	60 (100%)
Sequential LITA, n (%)	6 (10%)
RITA	-
Radial graft	-
Time in the operating room (h)	3.9±0.9#
Bypass time (min)	92.7±25.7#
Clamping time (min)	83±26.4#

LITA=left internal thoracic artery; AIVA=anterior interventricular artery; RITA=right internal thoracic artery

# mean ± standard deviation;

$ median ± interquartile range (p25-p75)

**Table 3:** Postoperative outcomes.

Variable	n = 60
ICU length of stay until extubation (h)	4 (3–5)$
Postoperative length of stay (days)	5 (4–6)$
Volume of liquid drained after 12 h (9)	300 (228–400)$
Dobutamine (mcg/kg)	___ $
Noradrenaline (mcg/kg)	0 (0–26)$
Creatinine peak (mg/dL)	1.1 (0.8–1.4)$
CK-MB peak (U/l)	49 (29–63) $
CPK peak (U/l)	664 (449–926)$
Lactate peak (mg/dl)	57 (39–72)$
Post-CPB TTPa (sec)	35 (31–39)$
Post-CPB INR	1.6 (1.5–1.7)$
Post-CPB platelets (10x3)	162 (128–195)$
Readmission, n (%)	1 (1.7%)
Respiratory infection	-
Wound infection	
Superficial	-
Urinary infection	-
Stroke	-
Atrial fibrillation, n (%)	5 (8.3%)
Reoperation	-
Renal dysfunction, n (%)	3 (5%)
Death, n (%)	1 (1.7%)

ICU=intensive care unit; CK-MB=creatine kinase-myocardial band; CPK=creatine phosphokinase; CPB= cardiopulmonary bypass

$ median ± interquartile range (p25-p75)

Table 4 shows data on arterial (aortic) tension as measured by A and B surgeons, intraluminal pressure, and cardioplegia line pressure following the sequence of repetitions of cardioplegia infusions.

**Table 4:** Pressures/tensions during cardioplegia infusions.

	Time point		
	First cardioplegia Median (p25–p75)	Second cardioplegia Median (p25–p75)	Third cardioplegia Median (p25–p75)
Surgeon A	55 (45–60)	60 (50–65)	60 (50–65)
Surgeon B	55 (45–60)	60 (50–65)	55 (45–66)
Intraluminal pressure (mmHg)	62 (56–68)	61 (56–66)	60 (54–64)
Cardioplegia line pressure (mmHg)	140 (130–150)	140 (126–140)	140 (129–150)

### Aortic wall tension - Agreement between surgeons A and B

**Fig. 1:**
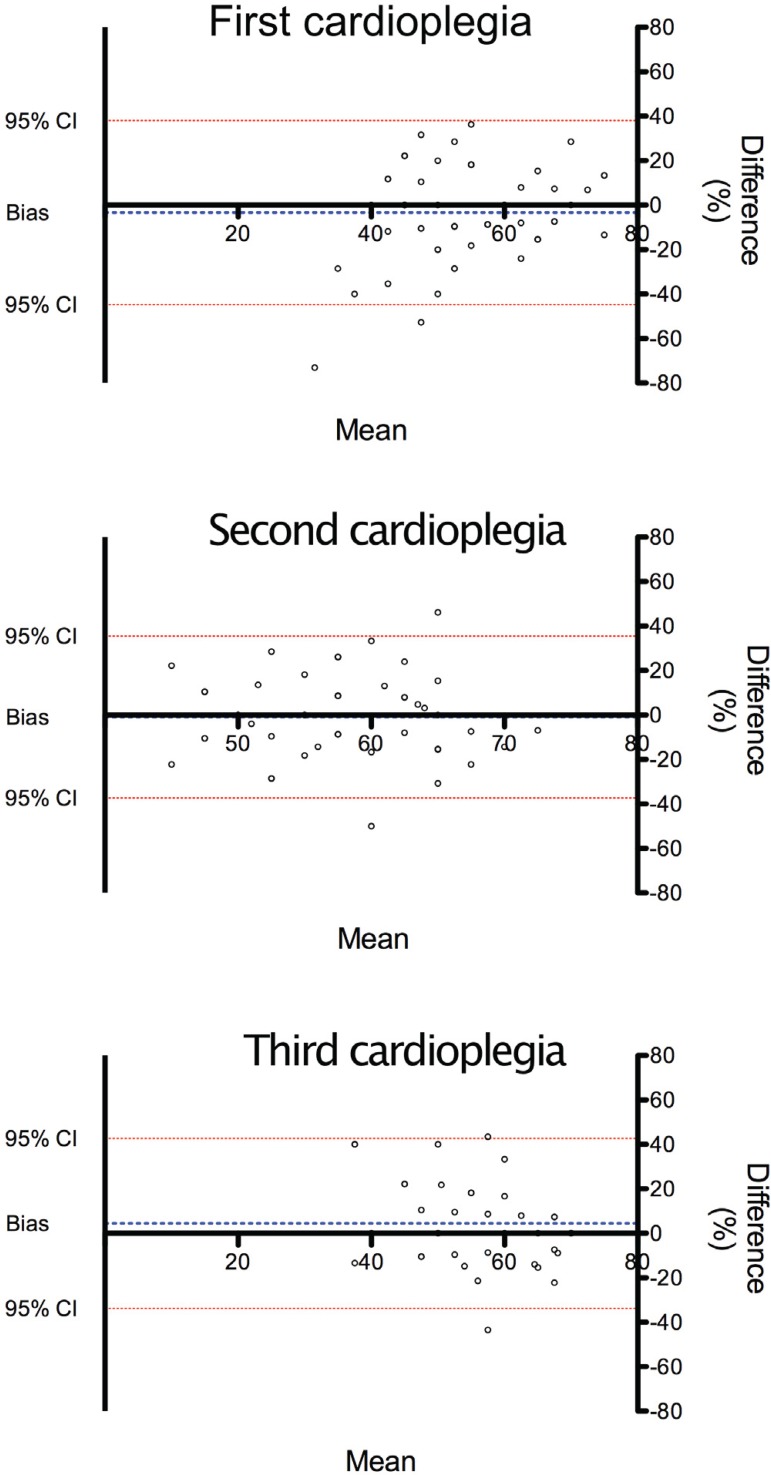
Bland-Altman plot comparing differences between the arterial tension measurements made by surgeons A and B.

Aortic wall tension during the first cardioplegia infusion had a bias of -3.4±21.11% (95% limits of agreement [LOA]: -44.79 to 37.98). Inter-surgeon differences (A-B) had skewness of -0.67 and kurtosis of 1.1, with normal distribution according to the Kolmogorov-Smirnov test. During the second cardioplegia infusion, bias was -0.9±18.58% (95% LOA: -37.35 to 35.46). Intersurgeon differences (A-B) had skewness of 0.07 and kurtosis of 0.11, with normal distribution according to the Kolmogorov-Smirnov test. During the third cardioplegia infusion, bias -0.9424±18.58% (95%LOA: -37.35 to 35.46). Intersurgeon differences (A-B) had skewness of 0.21 and kurtosis of 0.05, with normal distribution according to the Kolmogorov-Smirnov test (Figure 1).

### Agreement between intraluminal pressure and mean aortic wall tension as measured by surgeons A and B

**Fig. 2:**
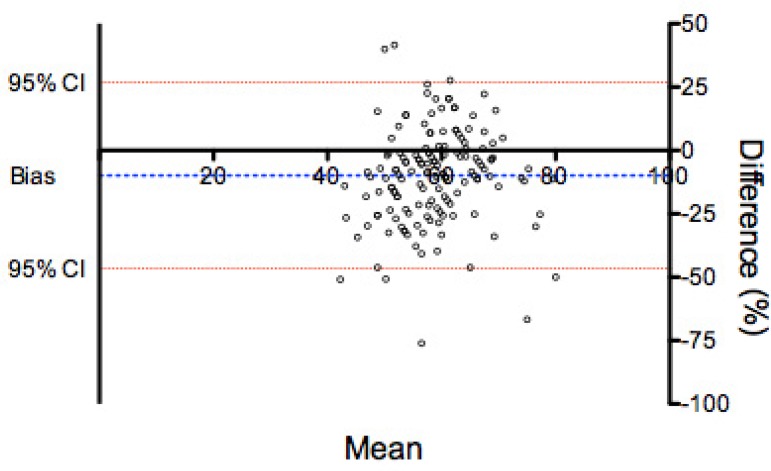
Bland-Altman plot comparing intraluminal pressure and mean aortic wall tension as measured by surgeons A and B.

**Fig. 3:**
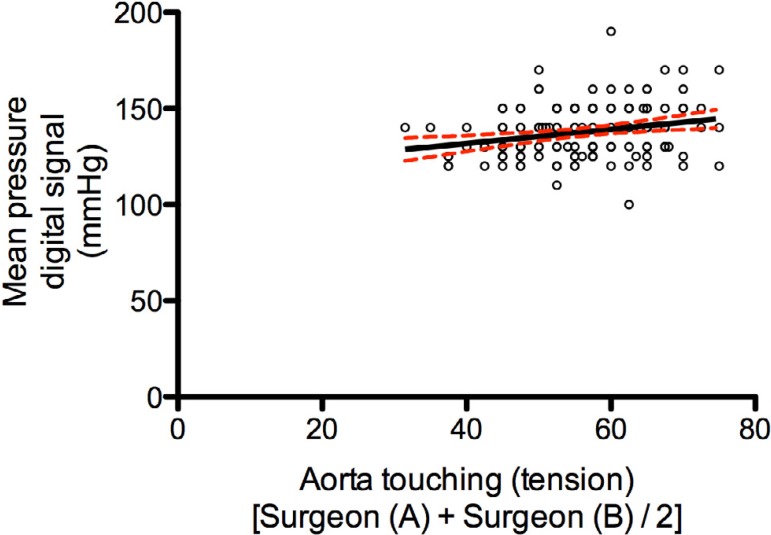
Spearman's rank correlation coefficient between intraluminal pressure and mean aortic wall tension as measured by surgeons A and B.

Mean values for each measurement of aortic wall tension (T) were calculated as follows: (T surgeon A + T surgeon B)/2. The agreement between these values and intraluminal pressure had a bias of -9.911±18.75% (95% LOA: -46.7 to 26.9). Differences between intraluminal pressure and mean aortic wall tension as measured by surgeons A and B had skewness of -0.3 and kurtosis of 1.0, with normal distribution according to the Kolmogorov-Smirnov test (Figure 2). The Spearman's rank correlation test found a weak correlation between the variables (r=0.25) (95% confidence interval [95%CI] 0.09-0.40); *P*=0.0021 (Figure 3).

### Correlation between intraluminal pressure and cardioplegia line pressure

**Fig. 4:**
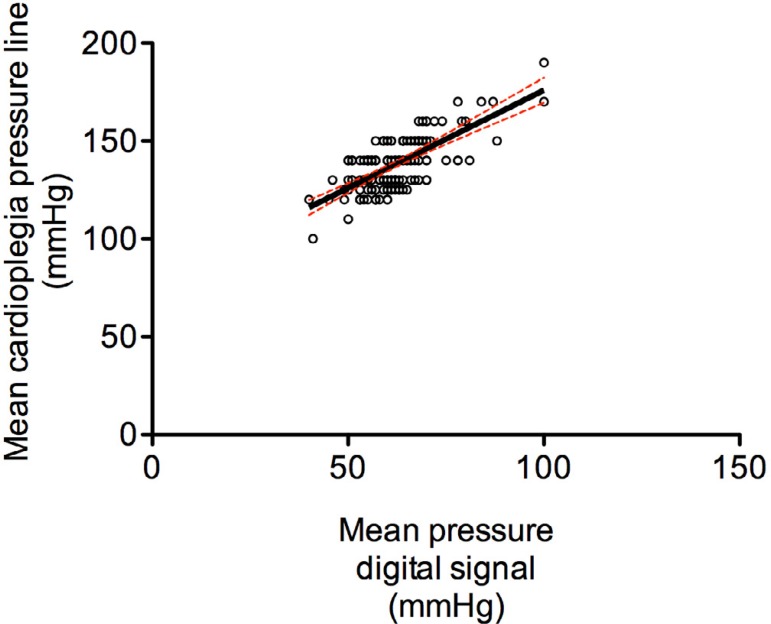
Spearman's rank correlation coefficient between intraluminal pressure and cardioplegia line pressure. Spearman's r=0.61 (95%CI 0.5-0.7); P<0.0001. R2=0.49; P<0.0001.

A moderate correlation was observed between intraluminal pressure and cardioplegia line pressure, with a Spearman's r=0.61 (95%IC; 0.5-0.7); *P*<0.0001 (Figure 4).

## DISCUSSION

Many authors have discussed the effects of the different types of cardioplegia^[9-11]^. Antegrade and/or retrograde delivery of cardioplegia has also been a matter of constant debate^[12-14]^. During retrograde delivery of solutions, there is usual concern with coronary sinus pressure because of its fragility and cases of rupture. With regard to antegrade administration, a recent survey showed that 95% of centers in the northeast United States usually measure cardioplegia line pressure^[15]^; however, this pressure is influenced by many interfering variables, such as circuit resistance due to its caliber and length, flow velocity, viscosity and temperature of the infused solution, and system afterload.

A study compared the direct measurement of intraluminal pressure with pressure estimation by aortic palpation and variation of cardioplegia line pressure^[16]^. As a result, the study showed that there was little correlation between measurements obtained directly and indirectly and that direct intravascular measurement is the most reliable method to determine aortic pressure during cardioplegic infusion and to ensure process effectiveness and safety. In agreement with Kato et al.^[16]^, our study found only a moderate correlation between cardioplegia line pressure and direct intraluminal measurement in the aortic root; therefore, cardioplegia line pressure should be used to investigate possible folds or obstructions during infusion, thus preventing accidents, such as circuit rupture resulting in risk of transmission of diseases due to inadvertent contact with blood, and avoiding high pressures usually associated with hemolysis.

The limitations of the study by Kato et al.^[16]^ lay in the fact that it assessed cardioplegia delivery in only 10 patients and that the methods of cardioplegia varied among these patients. Some patients were given an antegrade infusion and others were given a retrograde infusion, methods that show a significant variation in infusion pressure. As in our study, the authors compared the measurement of aortic arterial tension by palpation and by intraluminal pressure. In line with their findings, our study did not find a significant correlation between these two measuring techniques and observed a possibly clinically acceptable bias but with very wide limits of agreement, which would lead to important inaccuracies when pressure is measured by palpation.

In addition to the divergence between measurements obtained by aortic palpation and by intraluminal examination, it is worth noting that the first method may be influenced by subjective confounding factors such as surgeon's experience. Our study found a small bias in interobserver comparison, but the presence of high LOA shows considerable variations between measurements.

The present study has some limitations, such as the lack of evaluation of the influence of aortic diameter on the result of the measurement of aortic tension by palpation and the fact that this study was not designed to assess postoperative outcomes related with the method used to control cardioplegia infusion pressure.

## CONCLUSION

The present findings reinforce that cardioplegia infusion should be controlled by measuring intraluminal pressure and that palpation and cardioplegia line pressure are inaccurate methods, and the latter should always be used to complement intraluminal measurement to ensure greater safety in handling of the cardioplegia circuit. These findings also support the inclusion of dual or triple lumen cannulas for antegrade cardioplegia delivery into the Brazilian public health system (SUS) materials list applied for cardiac surgeries in Brazil performed in the Public Health System, which certainly account for the majority of cases operated in this country.

**Table:** 

Authors' roles & responsibilities	
JBL	Substantial contributions to the conception or design of the work; or the acquisition, analysis, or interpretation of data for the work; drafting the work or revising it critically for important intellectual content; agreement to be accountable for all aspects of the work in ensuring that questions related to the accuracy or integrity of any part of the work are appropriately investigated and resolved; final approval of the version to be published
CCMSJ	Substantial contributions to the conception or design of the work; or the acquisition, analysis, or interpretation of data for the work; drafting the work or revising it critically for important intellectual content; agreement to be accountable for all aspects of the work in ensuring that questions related to the accuracy or integrity of any part of the work are appropriately investigated and resolved; final approval of the version to be published
